# Barriers to Antiretroviral Therapy Adherence Among Children in Ekurhuleni, South Africa: A Descriptive Study

**DOI:** 10.3390/ijerph23050623

**Published:** 2026-05-08

**Authors:** Palesa Sokazi, Zelda Janse van Rensburg, Wanda Jacobs

**Affiliations:** Department of Nursing, Faculty of Health Science, Doornfontein Campus, University of Johannesburg, 55 Beit St., Doornfontein, Johannesburg 2028, South Africa; sokazi.palesa@gmail.com (P.S.); wandaj@uj.ac.za (W.J.)

**Keywords:** adherence, anti-retroviral therapy, children, HIV, South Africa

## Abstract

**Highlights:**

**Public health relevance—How does this work relate to a public health issue?**

- South Africa’s status as the country with the highest HIV prevalence globally places an enormous burden on the public health system, particularly in relation to prevention, treatment, and long-term care. Children living with HIV represent a vulnerable population whose health outcomes directly influence progress toward national and global HIV targets, including viral suppression and reduced HIV-related morbidity and mortality.
- Children’s adherence to antiretroviral therapy (ART) is largely dependent on their parents, caregivers, or guardians (PCGs), making ART adherence not only an individual health issue but a household- and community-level concern. Inadequate support or challenges faced by PCGs can undermine treatment effectiveness, increasing the risk of viral rebound, drug resistance, and onward transmission, all of which have significant public health consequences.

**Public health significance—Why is this work of significance to public health?**

- ART adherence below optimal levels threatens the effectiveness of HIV treatment programmes by increasing the risk of virological failure, disease progression, and the development of drug-resistant HIV strains. These outcomes can undermine national efforts to control the HIV epidemic and place additional pressure on already constrained public healthcare resources through increased hospitalisations, need for second-line therapies, and long-term care costs.
- The barriers experienced by parents, caregivers, and guardians (PCGs) in understanding HIV- and ART-related blood results, combined with missed appointments, poor follow-up, and forgotten doses, point to critical gaps in health literacy and continuity of care. These barriers compromise viral suppression in children, increasing the likelihood of ongoing HIV transmission later in life and reducing the overall effectiveness of public health HIV interventions.

**Public health implications—What are the key implications or messages for practitioners, policy makers and/or researchers in public health?**

- For practitioners and policy makers, strengthening caregiver-focused health literacy interventions should be a public health priority. Tailored, culturally appropriate education for PCGs on ART adherence, interpretation of HIV-related blood results, and the importance of follow-up care can improve viral suppression among children. Integrating caregiver support into routine paediatric HIV services may reduce treatment failure, drug resistance, and long-term healthcare costs.
- Research: HIV outcomes of children should be addressed through household- and community-level strategies rather than child-focused interventions alone. There is opportunity for future research keeping this in mind. Policies that support differentiated service delivery, reminder systems, and continuity of care models along with further research into caregiver barriers and support needs should be encouraged for future research.

**Abstract:**

This study aimed to explore and describe the barriers to Antiretroviral Therapy (ART) adherence among children in Ekurhuleni, Gauteng. A quantitative, cross-sectional design using a survey method was employed. Convenience sampling was used to recruit 157 parents, guardians, and caregivers (PGCs) who consented to participate in the study. Data was collected using self-report questionnaires and analysed using descriptive statistics and frequency distributions. The study was not designed or statistically powered to formally test associations between variables; therefore, only descriptive statistical analyses were conducted. The reliability and validity of the instrument were ensured, and ethical clearance was obtained from the relevant authorities prior to data collection. The study was conducted in accordance with established ethical principles and in compliance with the Declaration of Helsinki. The findings revealed that there were multiple barriers to children’s adherence to ART. Approximately one-third of PGCs reported being fully informed about the importance of ART adherence, while the majority indicated being only partially informed. Missed doses emerged as a significant challenge, with a substantial proportion reporting missed medication on one or more days, and only 31.2% administering ART consistently on time. Difficulties in understanding blood test results were also reported. In addition, a notable proportion of PGCs admitted to missing clinic appointments. These findings emphasize the need for strengthened caregiver education, ongoing support, and tailored interventions directed at primary health care nurses to promote consistent ART adherence among children.

## 1. Introduction

Human immunodeficiency virus (HIV) attacks the immune system, specifically CD4 cells, weakening immunity and increasing vulnerability to opportunistic infections such as tuberculosis and cryptococcal meningitis [1]. In children, HIV poses an even greater challenge due to the immaturity of their immune systems. Globally, an estimated 2.73 million children were living with HIV in 2021, of whom 300,000 were newly infected and 120,000 died from AIDS-related causes [2]. South Africa (SA) carries the highest burden of HIV worldwide, with approximately 7.8 million people living with the virus and 50,000 deaths reported in 2023 [3]. Among these, 238,000 are children, yet only about half are receiving treatment [4]. The Thembisa model estimates indicate that new infections remain high, with 149,000 in 2023, and the total number of people living with HIV is projected to continue rising [5]. This persistent high burden places children at increased risk of HIV exposure, particularly through mother-to-child transmission during pregnancy, delivery, or breastfeeding [6]. As the number of people living with HIV grows, more children face the dual challenge of being infected themselves or becoming vulnerable due to illness or loss of their caregivers, further contributing to the social and health impacts of the epidemic.

In response to the continued rise in HIV infections in SA, a range of prevention and treatment programmes have been implemented to reduce transmission and improve health outcomes of children. The Prevention of Mother-to-Child Transmission (PMTCT) programme is one of the most critical interventions for children, as it reduces the likelihood of HIV transmission during pregnancy, labour, delivery, and breastfeeding. With effective ART provided to HIV-positive mothers and their infants, Mother to Child Transmission (MTCT) rates can be reduced to below 5% [1]. Similarly, Pre-Exposure Prophylaxis (PrEP) is offered to HIV-negative individuals at high risk of infection, while Post-Exposure Prophylaxis (PEP) provides a short-term course of ART to individuals following possible exposure, such as sexual assault or occupational injury [1]. These programmes play a vital role in protecting both children and adults, thereby reducing the long-term burden of HIV. Complementing these prevention strategies, the UNAIDS “95-95-95” targets were introduced to accelerate progress in controlling the epidemic. These targets aim for 95% of all HIV-positive people to know their status, 95% of those diagnosed to receive sustained ART, and 95% of those on treatment to achieve viral suppression by 2030 [7]. Achieving these goals is especially important for children, as early diagnosis, timely initiation of ART, and consistent viral suppression are critical to improving survival, supporting normal growth and development, and reducing HIV-related morbidity and mortality.

Children living with HIV are particularly vulnerable because their immune systems are still immature, making them less able to fight infections and more likely to experience rapid disease progression [8]. Compared to adults, HIV-positive children often present with higher viral loads, faster CD4 cell depletion, and an increased risk of opportunistic infections such as pneumonia, tuberculosis, and diarrheal diseases [9]. In addition to physical health risks, HIV has been shown to adversely affect neurodevelopment, with studies reporting poor cognitive outcomes, language delays, and difficulties in school performance [10]. These health challenges not only compromise children’s survival but also impact their social and emotional development, further increasing their vulnerability.

Given these risks, the protection and care of children living with HIV are not only a pressing public health priority but also an ethical and constitutional obligation. Effective treatment is essential to safeguarding their survival, growth, and overall well-being. ART remains the cornerstone of pediatric HIV management, as it suppresses viral replication, preserves immune function, and reduces the incidence of opportunistic infections [6]. The success of ART, however, depends on early initiation and the selection of appropriate drug combinations [11]. Strict adherence to treatment regimens is critical, as it determines whether therapeutic goals are achieved.

Adherence is broadly defined as the extent to which patients take medications as prescribed by healthcare providers [12,13]. Research consistently demonstrates that maintaining adherence levels above 90% is strongly associated with sustained viral suppression, reduced risk of developing drug-resistant strains of HIV, improved immune recovery, and enhanced quality of life [14]. In contrast, poor adherence to ART compromises treatment outcomes and can result in treatment failure. Poor adherence also increases the risk of recurrent opportunistic infections and preventable hospitalizations among HIV-infected children. In addition, poor adherence further raises healthcare costs and contributes to drug resistance, threatening the long-term effectiveness of ART programmes [15].

Poor adherence to ART among children is influenced by a complex interplay of parent, guardian and caregiver (PGC)-related, child-related, health system, and socio-economic barriers. Recent evidence indicates that caregiver knowledge gaps, limited health literacy, and inadequate understanding of viral load monitoring significantly contribute to missed doses and inconsistent medication administration [1]. Stigma and fear of disclosure remain persistent barriers, particularly in school settings and extended family environments, where PGCs may avoid administering medication openly to prevent discrimination [16,17]. Socio-economic challenges such as poverty, food insecurity, transport costs to clinics, and caregiver unemployment further undermine consistent adherence, especially in resource-limited communities [18,19]. Health system barriers, including long waiting times, medication stock-outs, negative attitudes of healthcare workers, and limited child-friendly ART formulations, have also been shown to affect adherence patterns [1,11]. A study conducted outside South Africa, in South America, Peru, identified similar barriers to ART adherence including side effects and a lack of support by caregivers [20].

Child-specific barriers also play a significant role. Younger children depend entirely on PGCs for medication administration, making adherence vulnerable to PGC fatigue or competing responsibilities. As children grow older, challenges such as pill burden, unpleasant taste of medication, treatment fatigue, and psychosocial distress become more prominent [21,22]. Adolescents transitioning toward independent medication management may experience denial, peer pressure, and mental health difficulties, which further compromise adherence [1]. Additionally, missed clinic appointments and difficulties understanding laboratory results, particularly viral load monitoring, have been associated with suboptimal adherence and delayed identification of ART treatment failure [2,23].

Therefore, promoting and supporting optimal ART adherence in children is not only vital for individual health outcomes but also for sustaining public health gains in the fight against HIV. This study therefore aimed to determine and describe the barriers to ART adherence among children in Ekurhuleni, Gauteng. The adherence of children on ART have experienced limited research attention. The findings of the study were expected to provide insight into the barriers, as reported by PGCs, that significantly influence children’s adherence to ART and that can be addressed through strengthened PGC education, ongoing support, and tailored interventions targeted towards PGCs to promote consistent ART adherence in children.

## 2. Materials and Methods

### 2.1. Study Design and Setting

We employed a quantitative, cross-sectional design with a survey method to determine and describe the determinants that influence ART adherence among children. Data collection was conducted between June and August 2023 in Ekurhuleni, Gauteng, an area serviced by 30 Primary Health Care (PHC) clinics. Five PHC clinics were purposively selected for the study as these five clinics offered a comprehensive range of HIV-related services, including HIV testing for children, initiation and management of ART for both children and adults, PMTCT, and the Expanded Programme on Immunization (EPI).

### 2.2. Study Participants

Our study population comprised 157 PGCs of children on ART residing in Ekurhuleni, Gauteng. Inclusion criteria required participants to be PGCs of children aged from birth to 14 years who were receiving ART services at one of the selected PHC clinics and who were directly responsible for administering the treatment to the child. A convenience sampling method was used to recruit 152 respondents who were readily accessible at the five clinics during the data collection period, allowing efficient enrolment of respondents. To account for potential non-response and spoiled questionnaires, the sample size was increased by 10%, resulting in a final target of 167 respondents. However, the response rate was lower than anticipated, with only 157 respondents completing the questionnaire, representing a 94% response rate.

### 2.3. Data Collection

Following approval from the relevant university structures and health authorities, the study was introduced to the designated gatekeepers. Clinic managers and nurses assisted by identifying PGCs who met the inclusion criteria and informing them about the study. The lead researcher subsequently met with interested PGCs to provide a detailed explanation of the study’s purpose, after which written informed consent was obtained. Those who were willing to take part were then issued with anonymous self-administered questionnaires (*see Supplementary Materials*) to complete. The self-constructed questionnaire was developed in English and consisted of closed-ended, multiple-choice questions. It started with demographic items, capturing the child’s age, gender, duration on ART, and caregiver (PGC) characteristics. Subsequent sections required respondents to select responses that best reflected their beliefs and concerns regarding ART and the administration of ART related to adherence. Clinic attendance was also enquired about. The final section focused on how HCPs influenced children’s ART adherence. Respondents completed the questionnaires while waiting for their child’s consultation and, upon completion, placed them in a sealed box located at the reception desk that was then collected by the lead researcher.

The questionnaire (*see Supplementary Materials*) was pre-tested at a PHC clinic in Ekurhuleni that was not part of the five clinics selected for the main study, ensuring that pre-test data were excluded from the final dataset. The pre-test was conducted with 12 respondents, and no modifications were required as the instrument was deemed valid and reliable for use.

### 2.4. Data Analysis

Upon completion of data collection, responses were entered into a Microsoft Excel spreadsheet for initial organization and cleaning. The dataset was then forwarded to a statistician for analysis. Statistical analyses were performed using IBM SPSS Statistics software (version 29). The findings were summarized using descriptive statistics and frequency distribution.

### 2.5. Ethics and Legal Considerations

The study adhered to the ethical principles of respect for participants’ information.

Autonomy, beneficence, non-maleficence, and justice outlined in the Helsinki Declaration were adhered to throughout the study. Ethical approval was obtained from the University of Johannesburg’s and the Gauteng Department of Health’s Research Ethics Committees. To ensure data security and confidentiality, the data was stored in a password-protected computer and only used for research purposes.

## 3. Results

### 3.1. Children’s and PGC s’ Characteristics (N = 157)

Regarding age, 29 children (18.5%) were between birth and five years, 68 (43.3%) were six to nine years, and 60 (38.2%) were ten to fourteen years old. Females comprised 60% of the sample, while males accounted for 40%. In terms of ART duration, only six children (3.8%) had been receiving treatment for less than six months, 23 (14.7%) for six months to one year, and the majority, 128 children (81.5%), had been on ART for more than a year.

Most PGCs, 93 (69.2%) were aged 18 to 35 years, 34 (21.7%) were 36 to 50 years, and 30 (19.1%) were 51 years or older. Among the PGCs, 12 (7.6%) were students, 86 (54.8%) were unemployed, and 59 (38%) were employed full-time. Only two (1.3%) were self-employed, 22 (14%) worked part-time, and 27 (17.2%) were retired. The relationship between the PGC and the child showed that 89 (56.7%) were the child’s own parent, 50 (31.8%) were a guardian to the child, and 12 (7.6%) were siblings of the child on ART. The remaining six (3.8%) were other caregivers who met the inclusion criteria. Regarding the number of children cared for, 52 respondents (33.1%) looked after one child, another 52 (33.1%) cared for three or more children, and 53 (33.8%) were responsible for two children. Respondents were also asked about their mode of transportation to the clinic. The majority, 149 (94.9%), reported walking, while only eight (5.1%) drove to the facility. None of the respondents reported using taxis. Table 1 shows the children and PCGs characteristics.

### 3.2. Beliefs and Concerns Regarding ART Adherence

Of the participants, 119 (75.8%) strongly agreed that their child’s health depended on ART, while 38 (24.2%) agreed. Only one caregiver (0.6%) expressed disbelief in the benefits of ART, whereas 137 (87.3%) strongly agreed and 19 (12.1%) agreed that ART is beneficial. Regarding concerns about administering ART, 10 (6.4%) strongly agreed and 52 (32.5%) agreed that giving ART to their children worried them, 25 (20.4%) were uncertain, 39 (24.8%) strongly disagreed, and 29 (15.9%) disagreed. In response to whether their child’s health would be impossible without ART, 52 (33.1%) strongly agreed, 53 (33.8%) were uncertain, and 52 (33.1%) disagreed. All respondents (157; 100%) were uncertain about concerns regarding the long-term side effects of ART. However, 154 (98%) strongly agreed and three (2%) agreed that ART is a lifelong treatment. Most respondents, 151 (96.2%), strongly disagreed that ART disrupted their children’s lives, while six (3.8%) agreed. A large proportion, 154 (98.1%), agreed that they sometimes worried about their child’s dependence on ART, and three (1.9%) were uncertain. Concerning ART side effects, 143 (91%) strongly disagreed with experiencing them, while seven (4.5%) were uncertain if the child experienced any side effects, and another seven (4.5%) agreed. Importantly, all respondents (157; 100%) strongly agreed that ART protects their children from becoming sick or worse. Respondents were asked whether they administered their child’s medication in the presence of others. The vast majority, 155 (98.7%), reported that they did not, while two respondents (1.3%) indicated that they did.

### 3.3. ART Administration and Adherence

Respondents were asked whether they could identify their child’s ART regimen. The majority, 122 (77.7%), were able to do so, while 35 (22.3%) were not consistently able to identify the treatment. Regarding the frequency of ART administration, 150 children (95.5%) took their medication once daily, while seven children (4.5%) took it twice daily. Respondents were asked if they were ever informed about the importance of ART adherence. A total of 97 (61.8%) reported being partially informed, 50 (32.8%) indicated they were fully informed, and 10 (6.4%) stated that they were not informed about the importance of adherence.

Regarding children’s adherence to ART in the last month, 69 respondents (43.9%) reported that no doses were missed, 43 (27.4%) indicated missing one day’s doses, 28 (17.8%) missed doses for two days, and 17 (10.8%) missed doses for three or more days. As a follow-up question, the respondents were asked about the ease of remembering to administer ART to their child. A slight majority, 81 (51.6%), reported finding it difficult to remember, while 76 (48.4%) indicated that they did not have trouble remembering. Respondents were asked whether they had ever stopped their child’s ART when the child was feeling better. None of the respondents reported doing so. When asked about timely administration of ART, 49 respondents (31.2%) reported always giving their child’s medication on schedule, while 108 (68.8%) indicated that doses were not consistently administered on time. Regarding ART side effects or a worsening of the child’s condition, 99 respondents (63.1%) indicated they would stop administering ART if the child’s condition worsened, while 58 (36.9%) stated they would continue treatment despite any deterioration.

### 3.4. Clinic Attendance

Respondents were asked about their child’s clinic visits over the past six months. The majority, 119 children (75.8%), attended more than four visits, 31 (19.7%) attended three visits, and seven (4.5%) attended two visits. Respondents were also asked about missed clinic appointments. A total of 88 children (56.7%) had not missed any appointments, 54 (34.5%) missed one appointment, 12 (7.6%) missed two appointments, and two (1.3%) missed three or more appointments. When asked about the convenience of clinic hours, all respondents (100%) reported being satisfied.

### 3.5. Healthcare Providers

Regarding healthcare providers, all respondents (157; 100%) agreed that healthcare providers (HCPs) greeted them upon arrival. When asked about interpretation of blood results, only 10 (6.4%) reported understanding the explanations provided, 31 (19.7%) were uncertain, while 59 (37.6%) strongly disagreed and 57 (36.3%) disagreed that their results were adequately explained. All respondents (100%) indicated that they were informed about the reason for their clinic visit. Regarding communication, 100 (63.7%) agreed and 46 (29.3%) disagreed that HCPs encouraged them to express their thoughts and concerns, with 11 (7%) remaining uncertain. Most respondents (146; 93%) agreed that HCPs listened and understood their input, while 11 (7%) strongly disagreed. In terms of information provision, 148 (94.3%) agreed that they received sufficient information, six (3.8%) strongly disagreed, and three (1.9%) were uncertain. All respondents (157; 100%) agreed that HCPs checked whether the treatment was acceptable to them, involved them in decision-making about their child’s treatment, and confirmed their understanding of the information provided. The majority, 147 (93.6%), agreed that HCPs addressed their concerns, while 3.2% were uncertain and 3.2% strongly disagreed.

## 4. Discussion

Most HIV-positive children are diagnosed at birth [24,25]. In South Africa, vertical transmission of HIV within the first two months of life has dramatically declined, from 23% in 2003 to just 0.7% in 2019 [26]. This notable reduction is largely attributed to expanded access to ART during antenatal care, the PMTCT programme, as well as the routine HIV testing of all pregnant women during antenatal care [26]. According to the South African clinical ART guidelines [11], ART for children should be initiated immediately following a positive PCR result, confirmed by a subsequent test.

In this study, all 157 children (100%) began ART immediately following a positive diagnosis. The child’s age corresponded with the duration of time they had been receiving ART, indicating timely initiation following diagnosis, as the reported treatment duration was consistent with each child’s age. Villiera et al. [27] suggested that the longer the child is on ART, the better the adherence to ART, which was also evident in our study. Also, in our study, more females were on ART than males; however, we did not find that the gender of the child had an association with ART adherence. The majority (59.2%) of the respondents were between 18 and 35 years old. The literature indicates that younger PGCs of children on ART may face additional challenges, such as putting personal goals on hold, failing to prioritize the child’s ART regimen and healthcare needs over competing demands, social pressures, and limited parental skills and knowledge [28]. Globally, it has been found that only one in three individuals aged 18–35 years demonstrates accurate knowledge of HIV prevention and management [29]. This gap in knowledge can have significant implications, as young adults often serve as primary PGCs in the household. Limited understanding of HIV transmission, treatment adherence, and preventive strategies may increase the risk of delayed ART initiation and adherence. Educational initiatives targeting younger PGCs could therefore be key to improving ART adherence rates in children [30].

A large proportion of PGCs demonstrated an understanding of the importance of ART for the child’s health and recognized that treatment is lifelong. This finding may reflect a generally positive perception of ART within the study population. While such perceptions could potentially support improved adherence, the cross-sectional nature of this study does not allow conclusions regarding causality. Despite this apparent awareness, several barriers related to ART adherence were identified. Many PGCs reported not being fully informed about the importance of consistent ART use, the consequences of missed doses, the interpretation of blood test results, and the importance of attending clinic appointments. Only about one-third of PGCs indicated that they were fully informed about ART adherence, while the majority reported being only partially informed. This finding suggests possible gaps in treatment education. Barriers to ART adherence may include limited consultation time, complex medical terminology, or communication barriers between healthcare providers and caregivers [31]. Although 43.9% of PGCs reported no missed doses in the previous month, a notable proportion indicated missing medication for one or more days, and only 31.2% reported consistently administering ART at the correct time. Previous research has shown that missed doses are associated with increased viral load, drug resistance, and poorer clinical outcomes in children [32]. Similar challenges have been reported in other African contexts. For example, a study conducted in Kenya found that 50% of children missed ART doses for more than 48 h [28]. Missed doses may be linked to practical challenges faced by PGCs, such as difficulty measuring doses accurately, unpleasant medication taste, and strict timing requirements. Younger children may also refuse medication or spit it out, further complicating adherence. Studies from Kenya and Namibia have additionally identified HIV-related stigma and low disclosure rates as factors associated with missed doses and reduced adherence [28,33]. Several PGCs reported difficulty understanding explanations of blood test results related to HIV and ART. Limited comprehension may be related to low health literacy, the use of medical jargon, or insufficient provider–PGC communication [34]. PGCs who struggle to interpret key indicators such as viral load and CD4 count may have difficulty fully understanding treatment progress and the importance of consistent adherence [35]. These findings underscore the importance of clear, simple, and culturally appropriate communication strategies to enhance PGCs’ understanding [35]. A notable proportion of PGCs also reported missing clinic appointments, which may affect continuity of care. Regular clinic visits are important for monitoring viral load and CD4 counts, assessing treatment response, identifying side effects, and detecting early signs of drug resistance [11]. Missed appointments may result in delays in medication refills, dose adjustments, and opportunities for adherence counselling. Over time, such disruptions could potentially compromise viral suppression and treatment outcomes [36,37]. Missed appointments may also reflect broader contextual factors, including transportation difficulties, caregiver workload, stigma, or limited understanding of the importance of follow-up care. While this study does not establish relationships or correlations, the findings highlight areas that warrant further investigation using a descriptive study design to better understand caregiver knowledge, treatment education, and pediatric ART adherence.

The study has some limitations. Its findings are contextual and may not be generalizable beyond the study setting. In addition, the sample size was relatively small (N = 157), which may limit the statistical power and representativeness of the results. Future research should consider larger, more diverse samples and incorporate objective measures of ART adherence, such as pharmacy refill records, pill counts, or electronic monitoring, to improve accuracy and generalizability. Another limitation of the study is that ART adherence was assessed through PGCs’ self-reports, which may be subject to recall bias and social desirability bias, potentially affecting the accuracy of the reported adherence levels particularly given that some primary caregivers demonstrated limited knowledge of ART and the challenges in administering medication. The absence of objective measures, such as pharmacy refill records or viral load suppression data, may therefore limit the validity of the adherence findings.

## 5. Conclusions

The findings in our study underscore that while South Africa has made impressive strides in early ART initiation and PGC engagement, adherence among children is influenced by several barriers, namely PGCs’ knowledge on the importance of ART adherence, understanding blood test results, missing doses and missing appointments. Strengthening the health education PGCs receive, especially for younger PGCs, improving support mechanisms for medication administration, enhancing communication about blood test results, and addressing stigma-related barriers are critical strategies for improving ART adherence among children. Addressing these barriers may not only improve individual health outcomes but also contribute to the broader goal of controlling the HIV epidemic among children in South Africa.

## Figures and Tables

**Table 1:** Characteristics of the children and the PGCs (N = 157).

Child’s Characteristics	Frequency (N)	Percentage (%)	Frequency (N)		Percentage (%)		Frequency (N)		Percentage (%)	Total	
										N	%
**Age**	**Birth** **–** **5 years**		**6–9 years**				**10–14 years**				
	29	18.5	68		43.4		60		38.2	157	100
**Sex**	**Females**			**Males**							
	94	60	63		40					157	100
**Number of years on ART**	**Less than 6 months**		**6–12 months**				**More than 12 months**				
	6	3.8	23		14.7		128		81.5	157	100
**PGC’s** **characteristics**	**Frequency (N)**	**Percentage (%)**		**Frequency (N)**	**Percentage (%)**		**Frequency (N)**		**Percentage (%)**	**Total**	
										**N**	**%**
**Age**	**18–35 years**			**36–50 years**			**51 years and older**				
	93	69.2		34	21.7		30		19.1	157	100
**Employment status**	**Employed**			**Unemployed**			**Student**				
	59	38		86		54.8	12	7.6		157	100
**Relationship with child**	**Child’s own parent**			**Guardian to the child**			**Sibling of the child**				
	89	56.7		50	31.8		12		7.6	157	100
**Number of children in care**	**One child**			**Two children**			**Three or more children**				
	52	33.1		52	33.1		53		33.8	157	100
**Mode of transport to clinic**	**Walking**			**Driving own vehicle**			**Taxi**				
	149	94.9		8	5.1		0		0	157	100

## Data Availability

The datasets generated during and/or analyzed during the current study are available from the corresponding author upon reasonable request.

## Supplementary Materials

The following supporting information can be downloaded at: https://www.mdpi.com/article/10.3390/ijerph23050623/s1, Department of Nursing Research Study Questionnaire.

## Abbreviations

The following abbreviations are used in this manuscript:

PGC	Parents, Guardians and Caregivers
ART	Antiretroviral therapy
HIV	Human immunodeficiency virus
PHC	Primary Health Care
