# Supplementary material for: Barriers to Antiretroviral Therapy Adherence Among Children in Ekurhuleni, South Africa: A Descriptive Study
Source: Int J Environ Res Public Health. 2026 May 8;23(5):623. doi: 10.3390/ijerph23050623 (PMC13206197; doi:10.3390/ijerph23050623)
Supplement: Supplementary file 1 [file ijerph-23-00623-s001.zip › ijerph-4116391-supplementary.pdf]

RESPONDENT CODE:	

UNIVERSITY OF JOHANNESBURG

DEPARTMENT OF NURSING

RESEARCH STUDY QUESTIONNAIRE

Factors impacting the adherence of antiretroviral therapy among children in Ekurhuleni, Gauteng, South Africa.

Dear Respondent

Thank you for your participating in this research and answering the following questions.

Please complete the questionnaire by using a (X) to the answer that is most relevant to you into the "box" next to the Question.

Section A: Demographic

1. How old is your child?

   (indicate the year and months of the age of your child, i.e. 5 years 3 months)

Year		months	

2. How long has your child been taking ART

Less than 6months	
6month to 1year	
More than 1year	

3. Gender of the child

Male		Female	

SECTION B: Caregiver characteristics

1. What is your age group?

18-25		26-30	
31-35		36-40	
41-45		46-50	
51 and older			

2. What is your current employment status

Student	
Unemployed	
Working full time	
Working part time	
Self-employed	
Retired	

3. With what type of transport do use to get to the clinic to collect medicine for your child?

Walking	
Driving	
Taxi	

4. What is your relation to the child?

Parent	
Sibling	
Guardian	
caregiver	
Other (please specify)	

5. How many children do you take care of?

1 child	
2 children	
More than 3 children	

Section C: Knowledge and beliefs about ART (ARV's)

Please indicate how strongly you agree or disagree with each of the following the statements

	Strongly agree	Agree	Uncertain	Strongly disagree	Disagree
My child's health presently depends on their antiretroviral therapy (also called ARV's)					
I do not believe in the benefits of ART					
Having to give my child ART worries me					
My child's life would be impossible without drinking ART					

I sometimes worry about the long-term effects of ART on my child					
I know that ART is lifelong					
ART disrupt my child's life					
I sometimes worry about my child becoming dependent on ART					
My child sometimes experiences side effects of ART					
ART protect my child from being sick or becoming worse					

Section D: ART Adherence

1. Can you identify your child's ART?

Yes		No		Not always	

2. How often do you give your child ART in a day

Once	
Twice	

3. Many people find it hard to administer the drug every single day. In the past 4 weeks how many days has the child missed taking all their doses

None		Two days	
One day		More than 3 days	

4. Do you sometimes find it difficult to remember administering ART to your child

Yes	
No	

5. When your child feels better, do you sometimes stop giving ART to your child?

Yes	
No	

6. Do you always give ART to your child at the correct time?

Yes	
No	

7. Sometimes when your child feels worse after taking ART, do you stop ?

Yes	
No	

8. How many appointments did the child have scheduled over the past 6 months?

One		Three	
Two		More than 4	

9. How many clinic appointments has the child missed over the past 6 months?

None		Two	
One		More than 3	

10. Number of visits recorded in your child cards

One		Three	
Two		More than 4	

11. Do you medicate your child in front others?

Yes	
No	

12. Is the clinic timing convenient for you?

Yes		No	

13. If no, please specify.

14. Have you been told about importance of adherence?

Yes		No		Partly	

15. How many pills does your child take per dose?

1 pill		2-3 pills		More than 3 pills	

Section E: Health system factors

1. Each statement below is about your relationship with the child's healthcare provider (HCP). This is the nurse or doctor that attended to your child today or when you come to the clinic. Please click on the one response that is most relevant. Please indicate how strongly you agree or disagree with each of the following the statements

	Strongly agree	Agree	Uncertain	Strongly disagree	Disagree
1.Your child's healthcare provider (HCP) greeted you and your child in a way that made you feel comfortable					
2.Your child's latest blood result was interpreted to you					
3.You understood what they explained to you about the results					
4.The HCP discussed your reason(s) for coming today					
5.The HCP encouraged you to express your thoughts concerning your child's health problems					
6.The HCP listened carefully to what you had to say and understood you					
7.The HCP gave you as much information as you wanted.					
8.The HCP checked to see if the treatment plan was acceptable to you and your child					
9.The HCP involved you in decisions about your child's health as much as you wanted					
10.The HCP responded to your questions and concerns.					
11.The HCP checked to be sure you understood everything.					

We have come to the end of this survey. We thank you for your time. We greatly appreciate your participation in this study.
